# Event and Comment

**Published:** 1910-12-15

**Authors:** 


### DENTAL REGISTER.

Vol. LXIV. December 15, 1910. No. 12

### EVENT AND COMMENT.

THE CLOSE OF THIS VOLUME AND THE PROMISE OF ANOTHER. With this issue the Dental Register completes another year and totals a continuous publication of sixty-four years without the lapse of a single issue. This is something of a record as it is the best made by any dental periodical. This Journal has had a remarkable experience in many ways; but its birth was fortunate in that it began as the organ of one of the first Dental Societies and had for its editors in its early history men of strong character and great professional ideals, for which they were willing to sacrifice. Its later history may not be specially characteristic as it has not been identified with any great movement. It has been content to serve in whatever manner seemed best as time has come and gone. During the past year we have tried to keep our readers in touch with the best that has been produced in our literature and we hope during the coming year to improve in this respect. In addition we are planning to present to our readers the subject of oral hygiene in a technical manner, as it seems to us the time is quite ripe for the profession to take up this work from the technical standpoint. The subject has been theoretically presented from various view points during the last three years, and it now seems that the profession and public ought to be ready to begin the serious work of rescue and preventive dentistry which has been all too long neglected. What we shall be able to accomplish in this cause will depend largely on the attitude taken by the profession toward this subject. With all the work done during the past year by the Oral Hygiene Committee of the National Dental Association and the various state and local societies, as well as the multiplication of Free Clinics in all the larger cities of the country it would certainly seem that the time was now ripe for the general installation of the work on a practicable basis in every dental office in the land. Just how this is to be done we do not know, but we believe if the profession was well coached in the technical details of prophylaxis treatment a great step would be taken toward a general acceptance of its value and necessity. With this ideal before us we expect to use much of our space the coming year in fostering this cause. We shall need and hope to have much co-operation from members of the profession who are already interested in this work; because it now needs the touch of experience to enliven the discussion and attract the attention of those absorbed in other lines of work from which they will not easily separate themselves. It takes no argument to convince those who have had any considerable experience in this work that it is the one great work of dentistry. It may be nesessary to organize these men into a society before the profession will realize the great importance of this work. This will be done if that is the only way it can be accomplished; but it would seem that we now have more than enough societies, and that with a general propaganda through the dental journals and in the societies already existing there should be no great obstacle in the way of arousing the profession to the necessity for taking up this work systematically and energetically. We direct our readers to two excellent papers in this issue on the treatment of Pyorrhea Alveolaris, by men who know from a wide experience the value of this rescue work. They are both well worth reading and preserving.

